# Pan-genome analysis of the R2R3-MYB genes family in *Brassica napus* unveils phylogenetic divergence and expression profiles under hormone and abiotic stress treatments

**DOI:** 10.3389/fpls.2025.1588362

**Published:** 2025-05-23

**Authors:** Haiyan Fan, Jiawei Li, Wencong Huang, Aoshuang Liang, Liqing Jing, Jintao Li, Qing-Yong Yang, Kede Liu, Zhiquan Yang

**Affiliations:** ^1^ National Key Laboratory of Crop Genetic Improvement, Huazhong Agricultural University, Wuhan, China; ^2^ College of Life Sciences, Xinyang Normal University, Xinyang, China; ^3^ Yazhouwan National Laboratory, Sanya, China

**Keywords:** R2R3-MYB, pan-genome analysis, phylogenetic relationship, plant hormones, abiotic stresses

## Abstract

**Introduction:**

The R2R3-MYB transcription factors (TFs) are pivotal regulators of plant growth, development, and stress responses. However, their genetic diversity and functional roles in *Brassica napus* remain underexplored at a pan-genome scale.

**Methods:**

We identified R2R3-MYB genes in 18 published rapeseed genomes and analyzed their genomic distribution patterns, gene duplication, selective pressure, gene structure, conserved motifs, and phylogenetic relationships using a pan-genome approach. Additionally, transcriptomic datasets from hormone treatments and drought/heat stress experiments were analyzed to identify hormone-responsive and stress-responsive genes.

**Results:**

We systematically identified 7,552 R2R3-MYB genes from 18 *B. napus* genomes, which were grouped into 353 gene clusters based on the pan-genome approach, including 139 core, 121 softcore, 68 dispensable, and 25 private gene clusters. Similar to Arabidopsis, the *B. napus* R2R3-MYB genes can be clustered into 29 subgroups based on the phylogenetic tree, suggesting conserved functional roles in *B. napus* and *A. thaliana*. *Cis*-element profiling highlighted enrichment in hormone-responsive and stress-related elements in the promoter regions of the R2R3-MYB genes. Transcriptomic analyses identified 283 hormone-responsive and 266 stress-responsive R2R3-MYB genes, and 30 co-regulated genes under drought and heat stress implicate their roles in combined stress adaptation.

**Discussion:**

These findings provide the first pan-genome resource for R2R3-MYB genes in *B. napus*, which can serve as pivotal targets for enhancing stress resilience in rapeseed breeding programs.

## 1 Introduction

The MYB family is one of the largest families of transcription factor (TF) in plants (Stracke et al., 2001; Dubos et al., 2010; Li et al., 2019). Typically, the members in the MYB superfamily can be categorized into four subfamilies based on the number of MYB domain repeats conferring the ability to bind DNA: 1R-MYB (1-repeat), R2R3-MYB (2-repeats), R1R2R3-MYB (3-repeats), and 4R-MYB (4-repeats) (Dubos et al., 2010). Each repeat consists of 50–53 amino acids that form three α-helices, with the second and third helices forming a helix-turn-helix (HTH) motif. Among them, the R2R3-MYB subfamily plays crucial roles in plant growth and development, metabolism, and responses to environmental stresses (Jung et al., 2008; Mandaokar and Browse, 2009; Jiang and Rao, 2020). Since the identification of the first plant R2R3-MYB gene, *COLORED1* (*C1*), which was demonstrated to be critical for anthocyanin biosynthesis in aleurone tissues in *Zea mays* (Paz-Ares et al., 1987), a substantial number of R2R3-MYB genes have been identified, such as 126 R2R3-MYB genes in *A. thaliana* (Dubos et al., 2010), 134 in V*itis vinifera* (Wong et al., 2016), 122 in *Camellia sinensis* (Wilkins et al., 2009), and 244 in *Glycine max* (Du et al., 2012). The R2R3-MYB gene family exhibits both diversity and conservation in plants (Du et al., 2015).

*Brassica napus* (AACC, 2n = 38) is an allotetraploid species originated from natural hybridization of *B. rapa* (AA, 2n = 20) and *B. oleracea* (CC, 2n = 18) approximately 7,500 years ago in the Mediterranean region (Chalhoub et al., 2014). In *B. napus*, the functions of several R2R3-MYB genes have been studied. *BnaMYB5* is specifically expressed in the seed coat and regulates seed coat color formation in *B. napus* (Dai et al., 2024). *BnaC2.MYB28* positively regulates the accumulation of glucosinolates (GSLs) in seeds (Zhou et al., 2023). *BnaMYB69* regulated biomass and disease resistance, and its downregulation enhances biomass and increases disease susceptibility by modulating the levels of phytohormones, chlorophyll, shikimate, and lignin (Lin et al., 2023). During the growth and development of rapeseed, it is frequently subjected to abiotic stresses such as drought, high temperature, salinity, and cold damage. These stresses often hinder the plant’s growth and development, resulting in a decline in both quality and yield. In addition, various hormones also regulate essential biological processes and responses to environmental stimuli. The identification of stress- and hormone-responsive genes in rapeseed has always been a crucial research direction (Liang et al., 2019; Zhang et al., 2022; Liu et al., 2023).

With the development of genome sequencing technologies, genome assemblies of multiple representative rapeseed varieties with different ecotypes and geographic origins have been released. In addition, advanced multi-omics technologies have made it possible to explore the diversity of MYB genes at pan-genome level (Chalhoub et al., 2014; Lee et al., 2020; Song et al., 2020; Davis et al., 2023; Qu et al., 2023; Wang et al., 2024; Wu et al., 2024; Zhao et al., 2024). In this study, we identify R2R3-MYB genes in 18 published rapeseed genomes and analyzed their genomic distribution patterns, gene duplication, selective pressure, gene structure, conserved motifs, and phylogenetic relationships using a pan-genome approach. Additionally, candidate genes responsive to various hormones and different stress conditions were identified. These findings provide important data resource for future functional studies and gene manipulation of R2R3-MYB genes in rapeseed breeding.

## 2 Materials and methods

### 2.1 Data sources

The 18 rapeseed genome sequences and annotations were collected from public databases. The genome assemblies of ZS11, Westar, No2127, Tapidor, Gangan, Zheyou7, Shengli and Quinta were retrieved from the BnPIR database (http://cbi.hzau.edu.cn/cgi-bin/rape/download_ext) (Song et al., 2021). The genome assemblies of Ningyou7 were retrieved from the BnPedigome (http://ibi.zju.edu.cn/bnpedigome/download.php) (Zou et al., 2019). The genome assemblies of Darmor was retrieved from the Genoscope (http://www.genoscope.cns.fr/brassicanapus) (Chalhoub et al., 2014). The genome assemblies of Xiaoyun was retrieved from the BnGDXY (https://yanglab.hzau.edu.cn/BnGDXY/#/download) (Wang et al., 2024). The genome assemblies of NTS57 was retrieved from the Rapeseed Cold Stress DB (http://oilseed.online:8000/Download/) (Wu et al., 2024). The genome assemblies of Express617 was retrieved from the Zenodo (https://doi.org/10.5281/zenodo.3524259) (Lee et al., 2020). The genome assemblies of DC1 was retrieved from the BnaOmics (https://bnaomics.ocri-genomics.net/dc1_genome) (Zhao et al., 2024). The genome assemblies of Da-Ae, GH06, ZY821, Xiang5A were retrieved from the National Center for Biotechnology Information (NCBI) (https://www.ncbi.nlm.nih.gov/) under the BioProject accession number PRJNA627442, PRJNA770894, PRJNA950196 (Davis et al., 2023; Li et al., 2023; Qu et al., 2023).

### 2.2 Identification of R2R3-MYB genes in *B. napus*

The Hidden Markov Model (HMM) profile representing the MYB_DNA-binding domain (PF00249) was downloaded from the Pfam protein family database (http://pfam.xfam.org/) (Finn et al., 2016). The amino acid sequences of 126 A*. thaliana* R2R3-MYB genes were first obtained from the Arabidopsis Information Resource (TAIR) database (https://www.arabidopsis.org/) (Dubos et al., 2010). Next, protein sequences of *A. thaliana* R2R3-MYB genes were used as queries to perform a BLASTP search (E < 1e^-10^) against the 18 *B. napus* genomes. HMMER (http://hmmer.org/download.html) was then employed to identify MYB_DNA-binding domains (PF00249) (E < 1e^-10^) as candidate R2R3-MYB genes (Potter et al., 2018). Moreover, NCBI-Conserved Domain Data (CDD) search (expectation value threshold = 0.01 and maximum hits = 500) (https://www.ncbi.nlm.nih.gov/Structure/cdd/wrpsb.cgi) and SMART (http://smart.embl-heidelberg.de) were used to further verify all identified R2R3-MYB genes in *B. napus.* Proteins failing to pass ​structural validation by both CDD and SMART, or lacking complete R2R3-MYB domain architecture, were excluded from subsequent analyses.

### 2.3 Phylogenetic analysis of R2R3-MYB genes

The physiochemical properties of R2R3-MYB proteins, including amino acids, molecular weights (MWs), isoelectric points (pIs), and grand average hydropathicity (GRAVY), were examined by employing the ProParam tool in the ExPASY website (https://web.expasy.org/protparam/) (Artimo et al., 2012). The subcellular localizations of R2R3-MYB proteins of *B. napus* were predicted using the WoLF PAORT online program (https://wolfpsort.hgc.jp/) (Horton et al., 2007).

### 2.4 Identification of orthologous gene groups

OrthoFinder (v2.4.0) (Emms and Kelly, 2019) was used to identify orthologous gene groups (OGGs) in 18 *Brassica napus* genomes with default parameters. OGGs were classified into four categories: core (present in all 18 accessions), softcore (present in 15–17 accessions), dispensable (present in 2–14 accessions), and private (present in only 1 accession). The longest protein from each OGGs was identified as the representative sequence.

### 2.5 Identification of the *cis*-elements of R2R3-MYB genes

To investigate *cis*-elements in the promoter regions, the 2000-bp genomic DNA sequences upstream of all R2R3-MYB genes in *B. napus* were analyzed using the PlantCARE database (https://bioinformatics.psb.ugent.be/webtools/plantcare/html/) (Lescot et al., 2002).

### 2.6 Chromosome distribution, duplication, and selection pressure analysis

The chromosomal distribution of R2R3-MYB genes in rapeseed was analyzed with the R package “ideogram”, using ZS11 genome as a representative. Gene duplication events were identified using the DupGen_finder (Qiao et al., 2019). To calculate *K*a/*K*s of each orthologous gene pair, the amino acid sequences were aligned using MUSCLE (Edgar, 2022), transformed into CDS alignments with PAL2NAL (Suyama et al., 2006), and then fed into the KaKs_Calculator (v2.0) (Wang et al., 2010).

### 2.7 Analysis of the structure and conserved sequences

The MEME program (https://meme-suite.org/meme/meme_5.3.3/tools/meme) (Bailey et al., 2015) was used to identify motifs in R2R3-MYB proteins in *B. napus*. Furthermore, the sequences of R2 and R3 repeats in R2R3-MYB proteins from *B. napus* and *A. thaliana* were aligned using ClustalX (Larkin et al., 2007). Sequence logos of the R2 and R3 repeats were then generated by TBtools (Chen et al., 2023).

### 2.8 Multiple sequence alignment and phylogenic analysis

The amino acid sequences of R2R3-MYB genes in *B. napus* were aligned using the ClustalW program (Thompson et al., 2003), and the unrooted neighbor-joining phylogenetic tree was constructed using MEGA 11.0 (Tamura et al., 2021) with the following parameters: Poisson model, pairwise deletion and 1,000 bootstrap replications. The ITOL website (https://itol.embl.de/) was used to display the phylogenetic tree (Letunic and Bork, 2024).

### 2.9 RNA-seq data and differential expression analysis

We collected and analyzed the transcriptome expression data of ZS11 from BnIR (https://yanglab.hzau.edu.cn/) (Yang et al., 2023) and BnTIR (Liu et al., 2021). The RNA-seq data from the ZS11 cultivar were obtained from leaves and roots under seven hormonal treatments (ABA, BR, ETH, GA, MeJA, tZ, IAA) at different time points (0.5, 1, 3, 6 h), and under six abiotic stress conditions (drought, freezing, cold, osmotic, salt, heat) at different time points (0.25, 0.5, 1, 3, 6, 12, 24 h), as well as expression data from nine different tissues (bud, cotyledon, leaf, petal, pollen, root, seed, silique, stem). Gene-level raw count data files were generated using featureCounts (Liao et al., 2014). The raw count data were imported into the Bioconductor package DESeq2 (Love et al., 2014) in the R language to identify differentially expressed genes (DEGs). The standard (|log2(fold change)| ≥1 and FDR-adjusted p-value < 0.05) was used to identify DEGs that were influenced by hormones or stresses at multiple time points.

DEGs were categorized as upregulated (genes upregulated at least at one time point), downregulated (genes downregulated at least at one time point), or complex (genes upregulated at one time point but downregulated at another). DEGs from the root and leaf datasets were merged for subsequent analysis.

## 3 Results

### 3.1 Distribution of R2R3-MYB genes in 18 *B. napus* genomes

**Figure 1:**
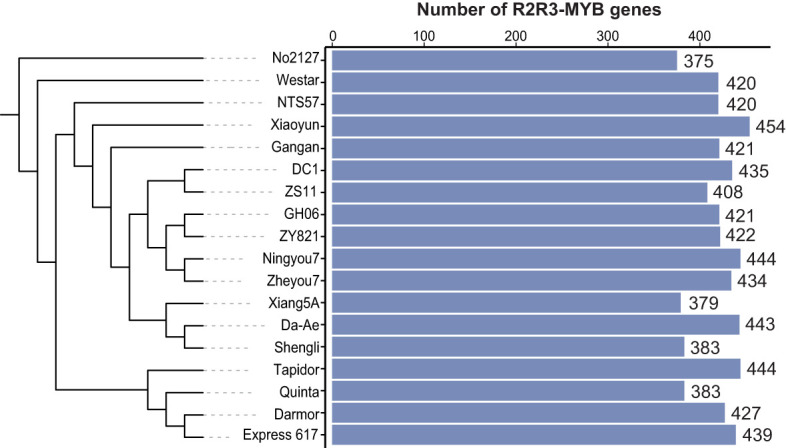
Number of R2R3-MYB genes in 18 *Brassica napus* genomes. The number of R2R3-MYB genes among each accession. Blue bars represent the number of R2R3-MYB genes in 18 genomes.

Considering the limitation of a single reference genome to capture the genetic information of a species (Tettelin et al., 2005; Sherman and Salzberg, 2020; Shi et al., 2023), we analyzed R2R3-MYB genes at a pan-genome level to better understand their genetic diversity. We collected the published genome assemblies and annotations of 18 *B. napus* accessions, including three spring accessions, nine semi-winter accessions, five winter accessions, and one synthesized accession (**Supplementary Table 1**). A total of 7,552 R2R3-MYB genes were identified across the 18 *B. napus* accessions by integrating multiple pipelines. The number of R2R3-MYB genes varied from 375 to 454 in individual *B. napus* genome, with an average of 420 genes per genome (**Figure 1A**; **Supplementary Table 2**).

**Figure 2:**
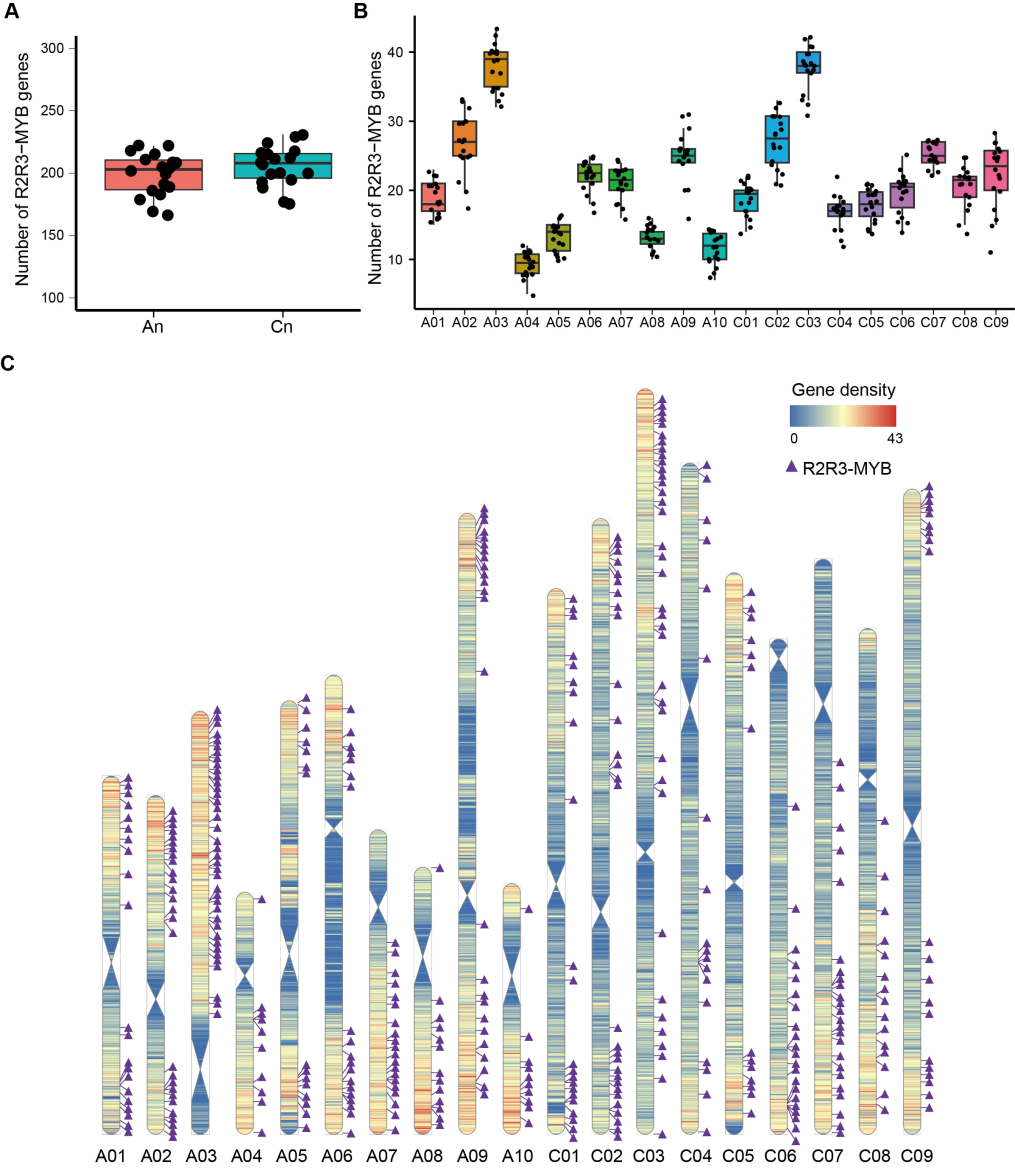
Distribution of R2R3-MYB genes across subgenomes and chromosomes in *Brassica napus.* **(A)** Number of R2R3-MYB genes in subgenomes An and Cn in the 18 genomes. Pink and blue boxes represent numbers of R2R3-MYB genes in subgenomes An and Cn, respectively. Black dots represent numbers of R2R3-MYB genes of 18 genomes. **(B)** Number of R2R3-MYB genes across chromosomes in 18 genomes. Boxes with different colors represent numbers of R2R3-MYB genes in A01-C09 chromosomes and black dots respresent numbers of R2R3-MYB genes of 18 genomes. **(C)** Chromosomal locations of R2R3-MYB genes in ZS11 genome. Blue to red regions in 19 chromosomes represent low to high gene density with the window size of 100 kb and the step size of 100 kb. Purple triangles represent R2R3-MYB genes.

The number of R2R3-MYB genes in the A and C subgenomes was similar (**Figure 2A**), indicating that both subgenomes contribute equally to the total count of R2R3-MYB genes. The R2R3-MYB genes were unevenly distributed on the chromosomes, with chromosomes A03 and C03 having the most (with an average number of 38 R2R3-MYB genes) and chromosome A04 having the fewest (with an average of nine R2R3-MYB genes) (**Figure 2B**).

Gene duplications, including whole genome duplication (WGD), tandem duplication (TD), proximal duplication (PD), transposon-mediated duplication (TRD), and dispersed duplication (DSD), are critical driving forces underlying gene family expansion in plant genomes (Flagel and Wendel, 2009; Qiao et al., 2019). We analyzed the sources of the R2R3-MYB genes in the 18 genomes. On average, 97.29% of the R2R3-MYB genes in individual genome are derived from WGD, which is higher than that observed at the whole-genome level (70.81%). These results indicate that WGD is the main source for the R2R3-MYB gene family in rapeseed (**Figure 2C**; **Supplementary Table 3**).

### 3.2 Orthologous group identification of R2R3-MYB genes

**Figure 3:**
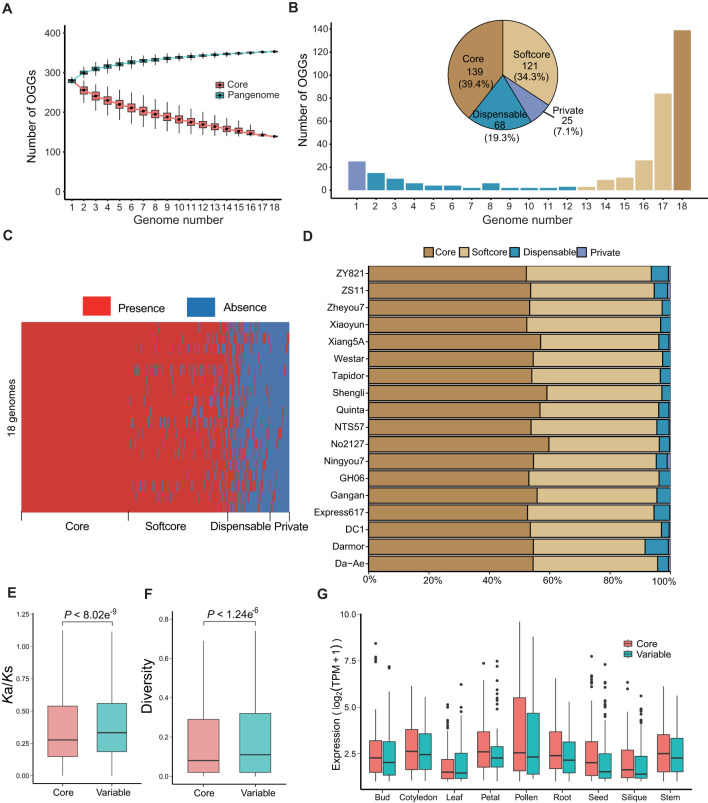
The panR2R3-MYB genes constructed from the 18 *Brassica napus* genomes. **(A)** The number of pan-genome and core R2R3-MYB OGGs in the 18 genomes. Blue and pink boxes represent pan-genome and core OGGs, respectively. **(B)** Composition of OGGs at the pan-genome level. The histogram shows the frequency distribution of OGGs shared by different numbers of genomes. The pie chart shows the proportion of different groups of OGGs. Deep yellow, light yellow, blue and purple bars and pies represent core, softcore, dispensable and private OGGs, respectively. **(C)** The presence and absence of R2R3-MYB OGGs in the 18 genomes. Red and blue regions represent present and absent OGGs, respectively. **(D)** Percentage of different R2R3-MYB gene groups in each of the 18 genomes. Deep yellow, light yellow, blue and purple bars and pies represent core, softcore, dispensable and private OGGs, respectively. **(E, F)** Comparison of *K*a/*K*s ratios **(E)** and sequence diversities **(F)** in promoters between core and variable OGGs (two-sided Student’s t test). **(G)** Expression levels of core and variable R2R3-MYB genes in ZS11 across various tissues: Bud, Cotyledon, Leaf, Petal, Pollen, Root, Seed, Silique, and Stem. In **(E–G)**, pink and blue boxes represent core and variable OGGs, respectively. .

Based on sequence similarity, 7,552 genes were clustered into 353 orthologous gene groups (OGGs). As the number of genomes increases, the number of R2R3-MYB OGGs exhibited a limited growth (**Figure 3A**), suggesting that the genomes we collected are sufficient to comprehensively represent the Pan-R2R3-MYB genes in *B. napus*. Based on the presence and absence of R2R3 MYB genes among the 18 rapeseed genomes, these OGGs were further classified into core genes (present in all 18 accessions), softcore genes (present in 15–17 accessions), dispensable genes (present in 2–14 accessions), and private genes (only present in one accession) (**Figures 3B, C**), with core genes (53.28%) and softcore genes (39.51%) being the major ones (**Figure 3D**), suggesting that most R2R3-MYB genes in *B. napus* are involved in regulating essential pathways. We renamed the 353 R2R3-MYB OGGs as Bna.MYB-C-001~139 (core), Bna.MYB-SC-140~260 (softcore), Bna.MYB-D-261~328 (dispensable), and Bna.MYB-P-329~353 (private) with a systematic pan-gene nomenclature (**Supplementary Table 4**). The *K*a/*K*s ratios of the core genes (*P* < 8.02e^-9^) and sequence variations in their promoters (*P* < 1.24e^-6^) were significantly lower than those of the variable (softcore, dispensable and private) genes (**Figures 3E, F**), indicating that core genes experienced stronger purifying selective pressure during evolution. In addition, compared to the variable genes, the core R2R3-MYB genes exhibit higher expression levels in all investigated tissues including bud, cotyledon, leaf, petal, pollen, root, seed, silique, and stem (**Figure 3G**), which is consistent with the results observed in other species at the whole-genome level (Cai et al., 2021; Li et al., 2024).

### 3.3 Phylogenetic tree and upstream regulatory sequence analysis of the R2R3-MYB genes

**Figure 4:**
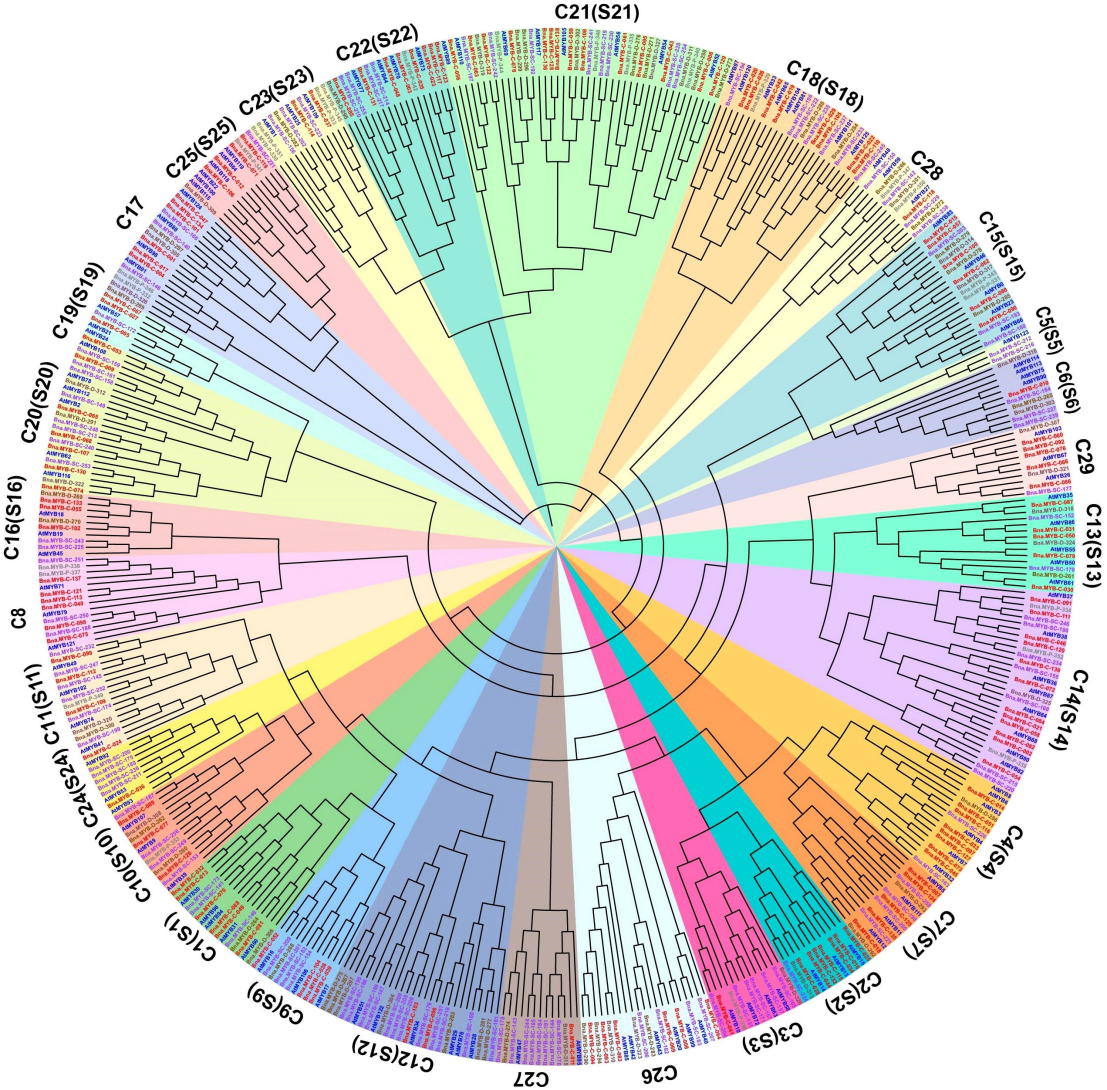
Phylogenetic classification of *A*. *thaliana* and *B*. *napus* R2R3-MYB genes. Representative R2R3-MYB genes for 353 OGGs were included. The R2R3-MYB genes in *B. napus* were classified into four categories: core (red labels), softcore (purple labels), and dispensable (brown labels) and private (grey labels), based on their presence in 18 *B. napus* genomes, with R2R3-MYB genes in *A. thaliana* highlighted in blue. The subfamilies of S1–S25 represented subfamilies based on groups of R2R3-MYB proteins in *A. thaliana* and subfamilies are highlighted in different colors accordingly.

To determine the functions of the R2R3-MYB genes in rapeseed, we investigated phylogenetic relationship between the R2R3-MYB genes in *A. thaliana* and *B. napus* genomes using the pan-genome gene family analyses (Tong et al., 2025). The longest protein was selected from each OGG as the representative, and aligned to that of 126 A*. thaliana* R2R3-MYB genes. Finally, a neighbor-joining phylogenetic tree was constructed. In the phylogenetic tree, the 353 R2R3-MYB OGGs were classified into 29 subgroups (**Figure 4**; **Supplementary Table 5**), consistent with the subgroup distribution observed in *A. thaliana* (**Supplementary Table 5**) (Stracke et al., 2001; Dubos et al., 2010), suggesting that the coding sequences of the *B. napus* R2R3-MYB genes may be similar to those of the *A. thaliana* ones in the same subgroup.

To further understand the functions of R2R3-MYB genes, we predicted *cis*-elements in the upstream 2 kb promoter regions of 7,552 R2R3-MYB genes. The identified *cis*-elements are associated with responses to light, plant growth and development, hormones, and abiotic stresses (**Supplementary Table 6**). Among the *cis*-elements related to responses to plant growth and development signals, the Opaque-2 site (O2-site) (31.14%) is the most abundant, which is associated with the tissue-specific expression in meristem and arginine metabolism, respectively (Chen et al., 2022). Among the *cis*-elements responses to plant hormones, the abscisic acid (ABA) responsive element ACGT-motif (ABRE) (36.27%), methyl jasmonate (MeJA) responsive elements including CGTCA-motif (18.45%) and TGACG-motif (18.45%) are the most abundant, while the remaining elements are salicylic acid, auxin, gibberellin, or other hormones responsive. The stress responsive elements mainly include drought-responsive elements (MYB binding site), anaerobic response element (ARE), low temperature response (LTR) and defense- and stress-response (TC-rich repeats).

### 3.4 Expression of R2R3-MYB genes under hormone treatments and abiotic stresses

**Figure 5:**
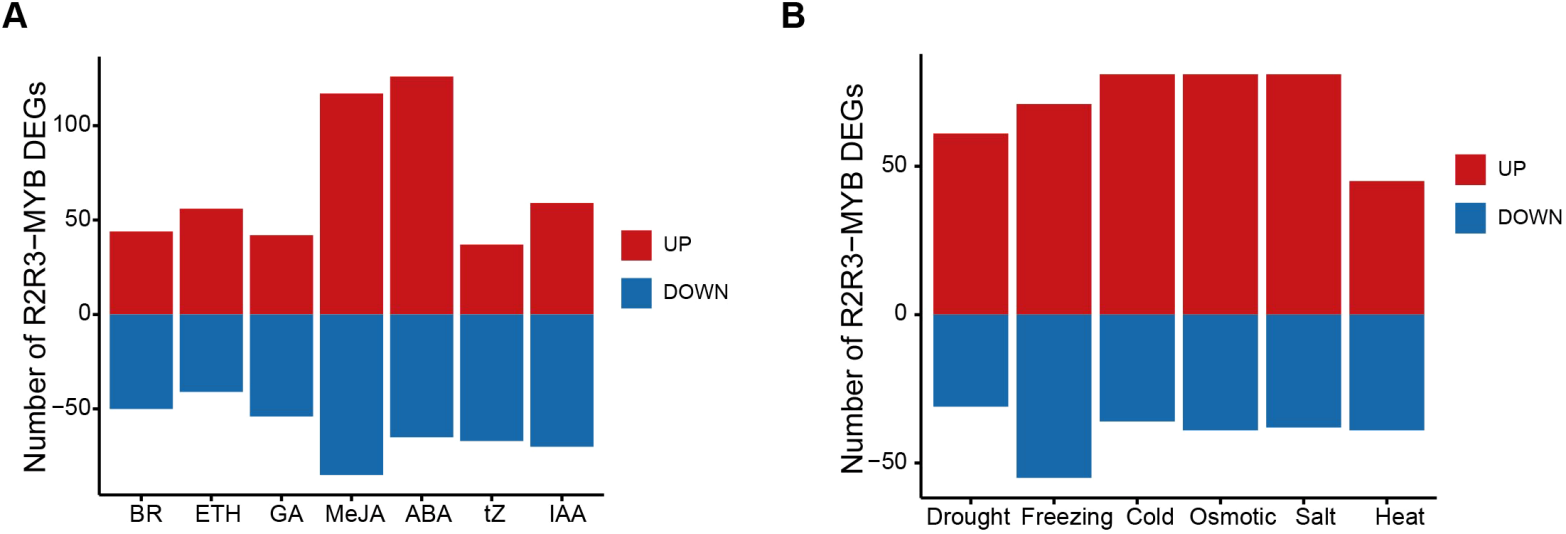
The number of up- and down-regulated DEGs under different hormone and abiotic stress treatments. **(A)** The number of up- and down-regulated DEGs under different hormone treatments. **(B)** The number of up- and down-regulated DEGs under different abiotic stress treatments. In **(A, B)**, red and blue bars represent up- and down-regulated DEGs, respectively. .

To identify potential roles in response to hormone treatments, we analyzed the ​transcriptome data from the ZS11 cultivar to investigate the expression patterns of the R2R3-MYB genes in response to ABA, ethylene (ETH), gibberellin (GA), indole acetic acid (IAA), brassinosteroids (BR), MeJA, and trans-Zeatin (tZ). Of 408 R2R3-MYB genes in ZS11 genomes, a total of 283 (69.4%) R2R3-MYB genes were differentially expressed under hormone treatment, compared to the control (**Supplementary Tables 7**, **8**). A total of 205, 102, 95, 117, 140, 221, and 106 DEGs were identified to be responsive to ABA, ETH, BR, GA, IAA, MeJA, and tZ, respectively (**Figure 5A**; **Supplementary Figures 1**, **2**, **Supplementary Table 9**). GA and ABA are essential endogenous regulators that primarily act antagonistically in plant growth, development, and environmental responses (Gómez-Cadenas et al., 2001; Liu and Hou, 2018). Thirty-four genes exhibited opposite changes in response to GA and ABA treatments (**Supplementary Table 10**). Previous studies in *A. thaliana* and *Oryza sativa* have shown that MeJA and ABA may synergistically regulate seed germination and drought responses (de Ollas and Dodd, 2016; Wang et al., 2020b). In *B. napus*, 80 genes were similarly regulated by MeJA and ABA, and these genes may play important roles in drought tolerance and seed germination. In addition, 47 DEGs only responded to one hormone, while 236 DEGs responded to two to seven different hormone treatments, suggesting extensive interactions between different plant hormones.

Given that R2R3-MYB genes are key regulatory factors in response to various abiotic stresses (Yang et al., 2012; Zhao et al., 2018; Gong et al., 2020), we further analyzed the ​transcriptome data from the ZS11 cultivar to compare their expression patterns under different abiotic stresses, including drought stress, freezing stress, cold stress, osmotic stress, salt stress, and heat stress (**Supplementary Table 11**). Totally, 266 (65.2%) R2R3-MYB genes were differentially expressed in response to abiotic stress treatments at least at one time point, compared to the control (**Supplementary Tables 11**, **12**), involving 143, 99, 126, 93, 206, and 203 DEGs under cold, drought, freezing, heat, osmotic, and salt stresses, respectively (**Figure 5B**; **Supplementary Table 13**). As cold and freezing conditions represent distinct intensities of low-temperature stress, 62 genes exhibited concordant expression patterns across both treatments (43 upregulated; 19 downregulated; **Supplementary Table 14**). This conserved transcriptional response implies these genes may orchestrate regulatory mechanisms underlying plant adaptation to thermal constraints. Abiotic stresses in nature are often not isolated, and combined stresses involving two or more factors are quite common (Yang et al., 2022). The combined stress of drought and heat can lead to a decrease in crop yield (Yang et al., 2022; Sato et al., 2024). Totally, 30 genes were identified to respond to both drought and heat stresses, suggesting these genes may play important roles under the combined stress of drought and heat.

The plant hormone ABA is known as the “stress hormone” due to its participate in response to abiotic stresses such as drought, cold, and salt (Wang et al., 2020a). Under drought, cold, and salt stress treatments, 76 (77%), 107 (74.8%), and 112 (81.2%) DEGs were differentially expressed under ABA treatment, respectively. This further indicates that ABA plays crucial roles in multiple stress responses.

### 3.5 Protein structure features of R2R3-MYBs

We further compared the physicochemical properties and structural features of the 7,552 R2R3-MYB genes, including protein length, molecular weight, and isoelectric point (**Supplementary Table 15**). The lengths of these R2R3-MYB proteins ranged from 107 to 957 amino acids, with an average of 311 amino acids. Most (89.2%) proteins have 200 to 400 amino acids. The molecular weight ranged from 12.06 kDa to 107.47 kDa, and the isoelectric point varied from 4.76 to 10.69. Among these, 57.8% proteins had isoelectric points below 7, classifying them as acidic proteins, while the remainder were basic proteins.

**Figure 6:**
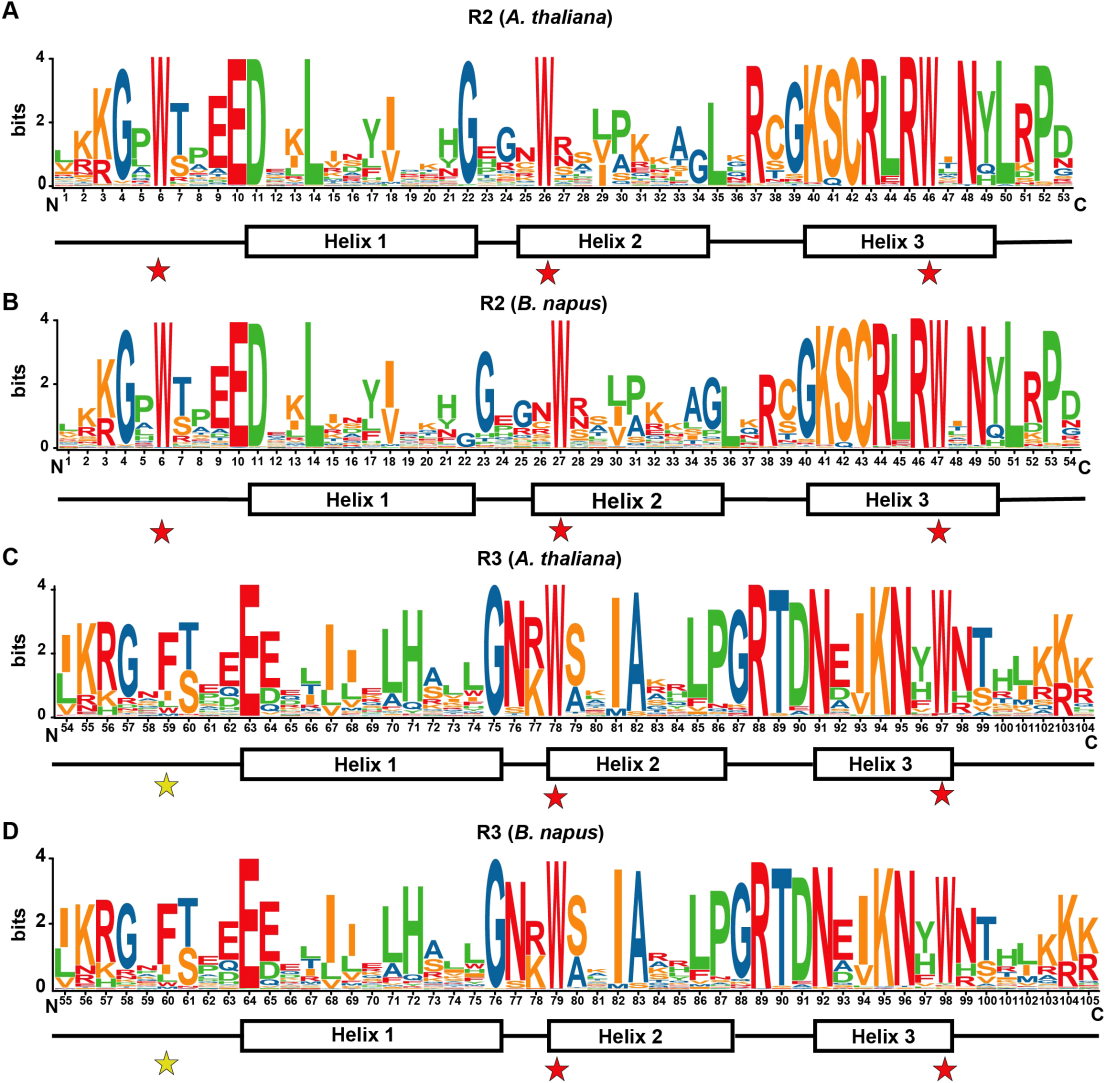
Sequence characteristics of the R2 and R3 repeats in R2R3-MYB proteins in *A. thaliana* and *B. napus*. **(A, B)** Sequence characteristics of the R2 repeats in R2R3-MYB proteins in *A. thaliana* **(A)** and *B. napus* **(B)**. **(C, D)** Sequence characteristics of the R3 repeats in R2R3-MYB proteins in *A. thaliana* **(C)** and *B. napus* **(D)**. The bit score represents the information content of each amino acid at specific positions within the sequence. The locations of the three α-helices are indicated (helices 1 to 3). Conserved tryptophan residues (Trp, W) within the MYB domain are highlighted with red asterisks, while substituted residues in the R3 repeat are denoted by yellow asterisks.

The R2 and R3 repeats within R2R3-MYB proteins display distinct amino acid patterns (**Figure 6**). The R2 repeat contains three regularly spaced and conserved tryptophan (Trp, W) residues, which stabilize three helices. In contrast, the R3 repeat features with only two conserved Trp residues, corresponding to the second and third Trp residues in the R2 repeat. The first Trp residue in the R3 repeat is often replaced by phenylalanine (F) and, less frequently by isoleucine (I) or leucine (L). An additional residue was identified after position 21 in the R2 repeats of rapeseed and Arabidopsis. Moreover, notable differences in residue type distribution were observed at positions 19, 20, 23, 33, and 36 in the R2 repeats and at positions 69, 71, and 73 in the R3 repeats. Consistent with previous studies (Dubos et al., 2010), helices 1 and 2 in the repeats exhibited lower conservation, while helix 3, due to its role in DNA recognition, was more conserved than helices 1 and 2. These findings indicate that the R2R3-MYB DNA-binding domain (DBD) in *A. thaliana* and *B. napus* is highly conserved, while also reflecting species-specific differences to some extent.

## 4 Discussion

The R2R3-MYB transcription factors play various biological roles, such as regulating plant growth, development, and responses to stresses (Dubos et al., 2010). In recent years, the functions of several R2R3-MYB genes have been preliminarily revealed in *B. napus* (Lin et al., 2023; Zhou et al., 2023; Dai et al., 2024). However, the functional mechanisms of many R2R3-MYB genes remain unclear (Luo et al., 2023). The number of genomes sequenced for the same plant species has experienced explosive growth over the past 20 years. In this study, we comprehensively analyzed the genomic distribution, gene structure, conservation of coding sequence, upstream regulatory sequences, and gene expression patterns of the R2R3-MYB genes in 18 *B. napus* genomes using a pan-genome approach. The pan-genomic analysis identified 7,552 R2R3-MYB genes from the 18 rapeseed genomes. The 7,552 R2R3-MYB genes were classified into 353 OGGs, which could be further classified into 29 subgroups (**Figure 4**; **Supplementary Table 5**). We noticed that dispensable OGGs are enriched in specific subgroups, such as C12 (S12), C17 (S17), and C21 (S21), suggesting that the amplification of R2R3-MYB genes may be biased towards the evolution of specific biological functions. The R2R3-MYB genes in the *B. napus* genome are mainly derived from WGD, and most R2R3-MYB genes have undergone purifying selection. Furthermore, the R2R3-MYB DBD domain is highly conserved and the *cis*-elements in the upstream regions of R2R3-MYB genes are associated with plant growth, development, and responses to stresses.

Plant hormones, including ABA, IAA, BR, CK, ETH, GA, and MeJA, are well-established regulators for plant growth and development, playing pivotal roles in mediating adaptive responses to diverse environmental conditions (Waadt et al., 2022). In this study, we identified R2R3-MYB genes that respond to seven distinct hormones and six different stress conditions based on hormone- and stress-related transcriptome analyses. Only 47 R2R3-MYB genes exhibited differential expression in response to a single hormone, indicating that the majority of the genes are involved in the response to multiple hormones. Similarly, merely 48 R2R3-MYB genes showed differential expression under a single stress condition, suggesting that most genes participate in the response to various stresses.

Previous studies indicated that ABA-responsive genes participate in the abscisic acid signaling pathway, thereby enhancing plant tolerance to abiotic stress (Uno et al., 2000). As for abiotic stress, the accumulation of abscisic acid was beneficial for plant to bolster their stress resistance (Xiong et al., 2002). In this study, 17 R2R3-MYB genes co-participate in response to abscisic acid and those activated under various stress conditions, suggesting that they may play important roles in the ABA-mediated response to environmental signals. The functions of these genes need to be further verified. Our study provides valuable insights into the interplay among plant hormones and their synergistic responses to abiotic stresses, which provides reference for future genetic breeding.

## 5 Conclusion

In this study, we systematically analyzed the members of the R2R3-MYB gene family in 18 *B. napus* genomes using a pan-genome approach. A total of 7,552 R2R3-MYB genes were identified, which were further classified into 29 distinct subgroups based on the phylogenetic tree. Comprehensive bioinformatic analyses were performed to investigate the physicochemical properties of the R2R3-MYB proteins, their motif composition, gene structures, and the prediction of cis-elements. Additionally, transcriptomic analysis was conducted to explore the expression patterns of these genes under various abiotic stresses and hormone treatments. These findings provide valuable insights into the role of the R2R3-MYB gene family in regulating plant responses to hormones and abiotic stresses. The extensive characterization of these genes offers potential candidate genes for future breeding programs aimed at improving stress tolerance and other agronomic traits. Furthermore, this study contributes to the broader field of pan-genomics by providing a reference for the study of gene families, enhancing our understanding of their evolution and functional diversity.

## Data Availability

The original contributions presented in the study are included in the article/**Supplementary Material**. Further inquiries can be directed to the corresponding author.
