# Supplementary material for: Pan-genome analysis of the R2R3-MYB genes family in Brassica napus unveils phylogenetic divergence and expression profiles under hormone and abiotic stress treatments
Source: Front Plant Sci. 2025 May 23;16:1588362. doi: 10.3389/fpls.2025.1588362 (PMC12141308; doi:10.3389/fpls.2025.1588362)
Supplement: Supplementary file 2 [file DataSheet2.pdf]

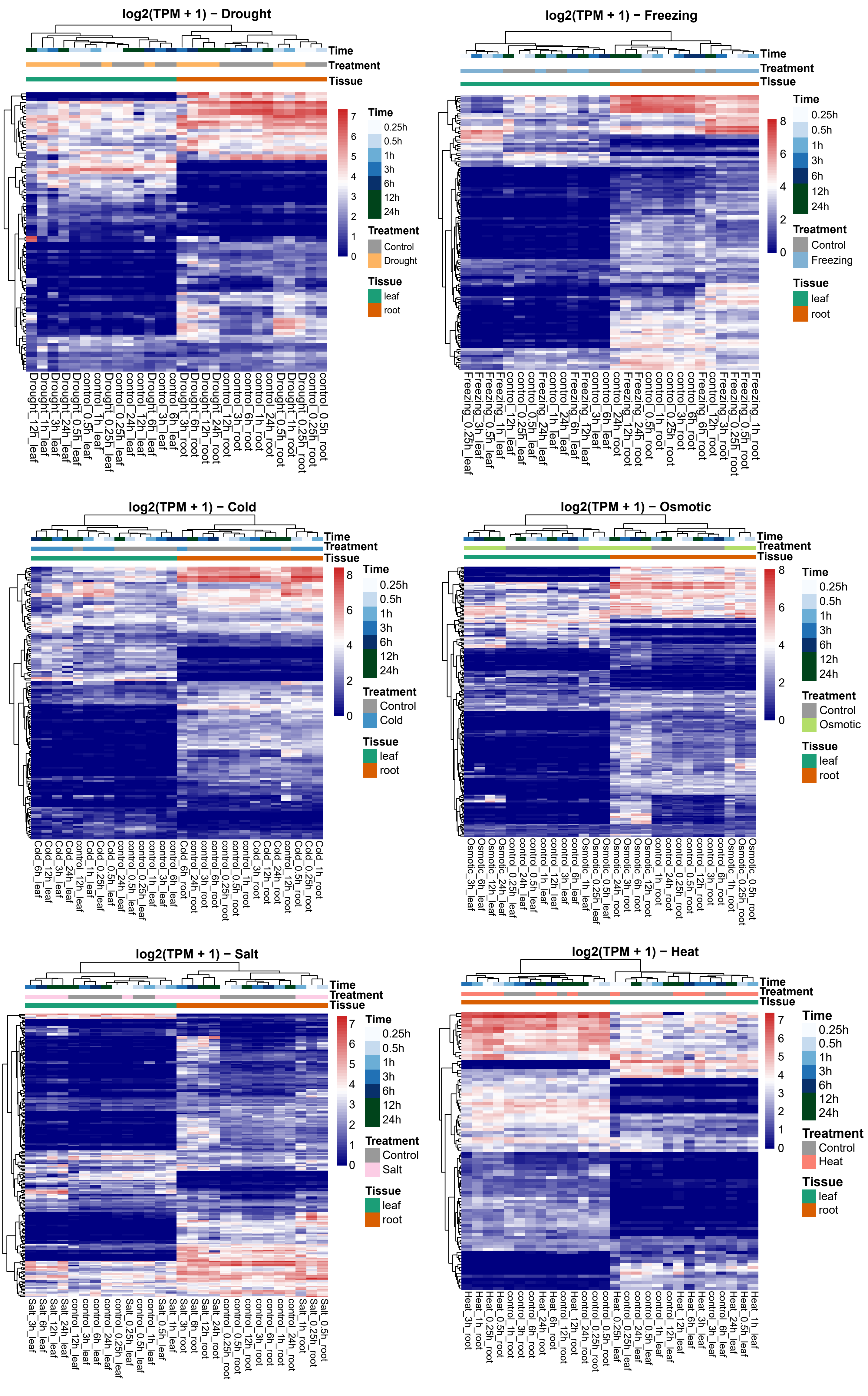

Figure S1: Expression patterns of differentially expressed R2R3-MYB genes under abiotic stresses. The heatmap color gradient from blue (low) to red (high) represents gene expression levels. Above the heatmap, three color bars are displayed. On the top bar, different colors indicate time points of abiotic stress treatments. In the middle bar, their colors represent stress-treated and control groups. On the bottom bar, their colors represent different tissues, including leaf and root.

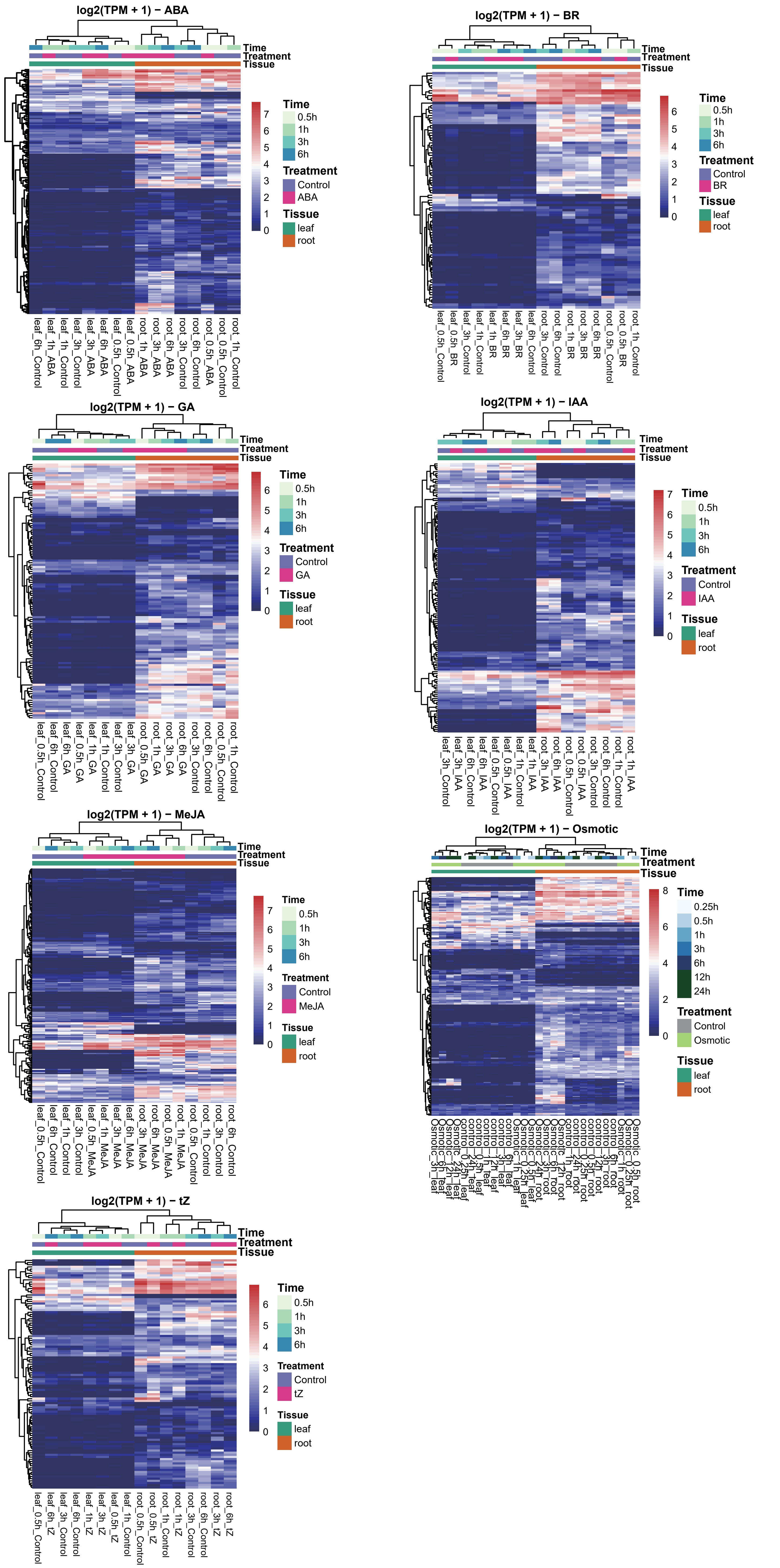

Figure S2: Expression patterns of differentially expressed R2R3-MYB genes under hormone treatments. The heatmap color gradient from blue (low) to red (high) represents gene expression levels. Above the heatmap, three color bars are displayed. On the top bar, different colors indicate time points of hormone treatments. In the middle bar, their colors represent hormone-treated and control groups. On the bottom bar, their colors represent different tissues, including leaf and root.
